# When the heart and hands tell a story: an intriguing case of Holt–Oram syndrome

**DOI:** 10.1186/s43044-024-00549-4

**Published:** 2024-09-02

**Authors:** Ilyas Atlas, Soukaina Zagdan, Mohamed Megzari, Salim Arous, Abdenasser Drighil

**Affiliations:** Cardiology Department, Ibn Rochd Hospital University, Casablanca, Morocco

**Keywords:** Holt–Oram syndrome, Genetic disorder, TBX5 gene

## Abstract

**Background:**

Holt–Oram syndrome is a rare genetic disorder caused by a mutation in the TBX5 gene, combining skeletal and cardiac malformations. Vital prognosis depends essentially on cardiac involvement, while skeletal malformations determine functional prognosis.

**Case presentation:**

We describe the case of a young patient aged 49, with no particular history, who presented to the emergency department with de novo congestive heart failure. Clinical examination revealed not only signs of heart failure, but also malformations such as triphalangia of the left thumb, prono-supination defects of both forearms and dorsolumbar scoliosis. The electrocardiogram showed that an atypical atrial flutter and transthoracic echocardiography revealed an atrial septal defect. We also performed a spinal scan to assess the severity of the scoliosis. Genetic studies confirmed a TBX5 gene mutation in the patient, and family screening revealed no similar cases in the family. Management consisted mainly of pharmacological treatment of heart failure, in addition to scoliosis management.

**Conclusion:**

Holt–Oram syndrome is a rare genetic disorder which should be suspected in the presence of any upper limb anomaly associated with cardiac malformation and confirmed by genetic study. A family investigation is necessary after diagnosis, because of autosomal dominant inheritance.

## Background

Holt–Oram syndrome is a rare genetic disorder that usually associates malformations of one or both upper limbs with congenital heart defects, as well as rhythm and/or conduction disorders requiring pacemaker implantation in certain situations [1]. This pathology is often due to an alteration in the sequence of the TBX5 gene located on chromosome 12 with an autosomal dominant mode of transmission [2]. During early cardiac development, TBX5 shows up to act basically as a transcriptional activator of qualities related to cardiomyocyte development and upstream of morphological signals for septation. In cardiac advancement, TBX5 is required for designing of the cardiac conduction framework and support of develop cardiomyocyte work [3]. Therefore, TBX5 gene mutation is often responsible of atrial or ventricular septal defects observed in Holt–Oram syndrome.

Our aim is to report the clinical, radiological, and genetic findings of this case in order to clarify the attitude to adopt toward this kind of patients.

## Case presentation

We describe a 49-year-old male patient, with no particular personal or family history, admitted to the cardiology department for acute rest dyspnea with ongoing palpitations. On admission, the patient was conscious 15/15, apyretic, normotensive to 124/91 and normocardial to 84 bpm, with a saturation of 96% on free air.

Physical examination found some clinical heart failure signs such as bilateral, symmetrical, soft edema of the lower limbs, taking the bucket at pressure and reaching mid-leg, there was also turgidity of the jugular veins with slight abdominal distention, indicating ascites. All these signs of right heart failure were associated with bilateral crepitating rales at the bases when auscultating the lung fields.

Cardiac auscultation revealed a systolic murmur localized at the pulmonary focus (upper left of the sternum) and a doubling of the second heart sound (B2), testifying to increased flow through the pulmonary valve.

**Fig. 1:**
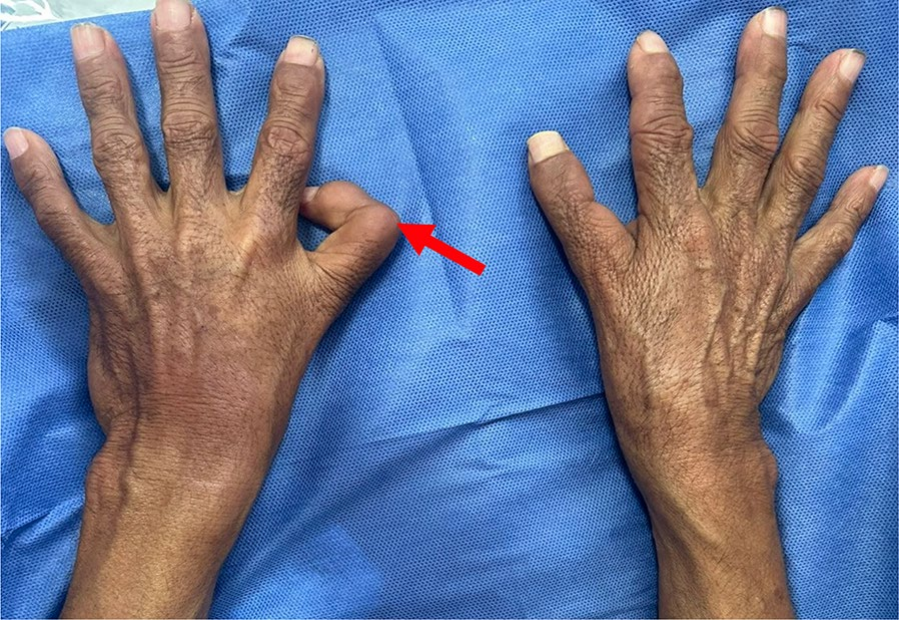
Photo of the patient's hands showing triphalangeal left thumb (red arrow)

**Fig. 2:**
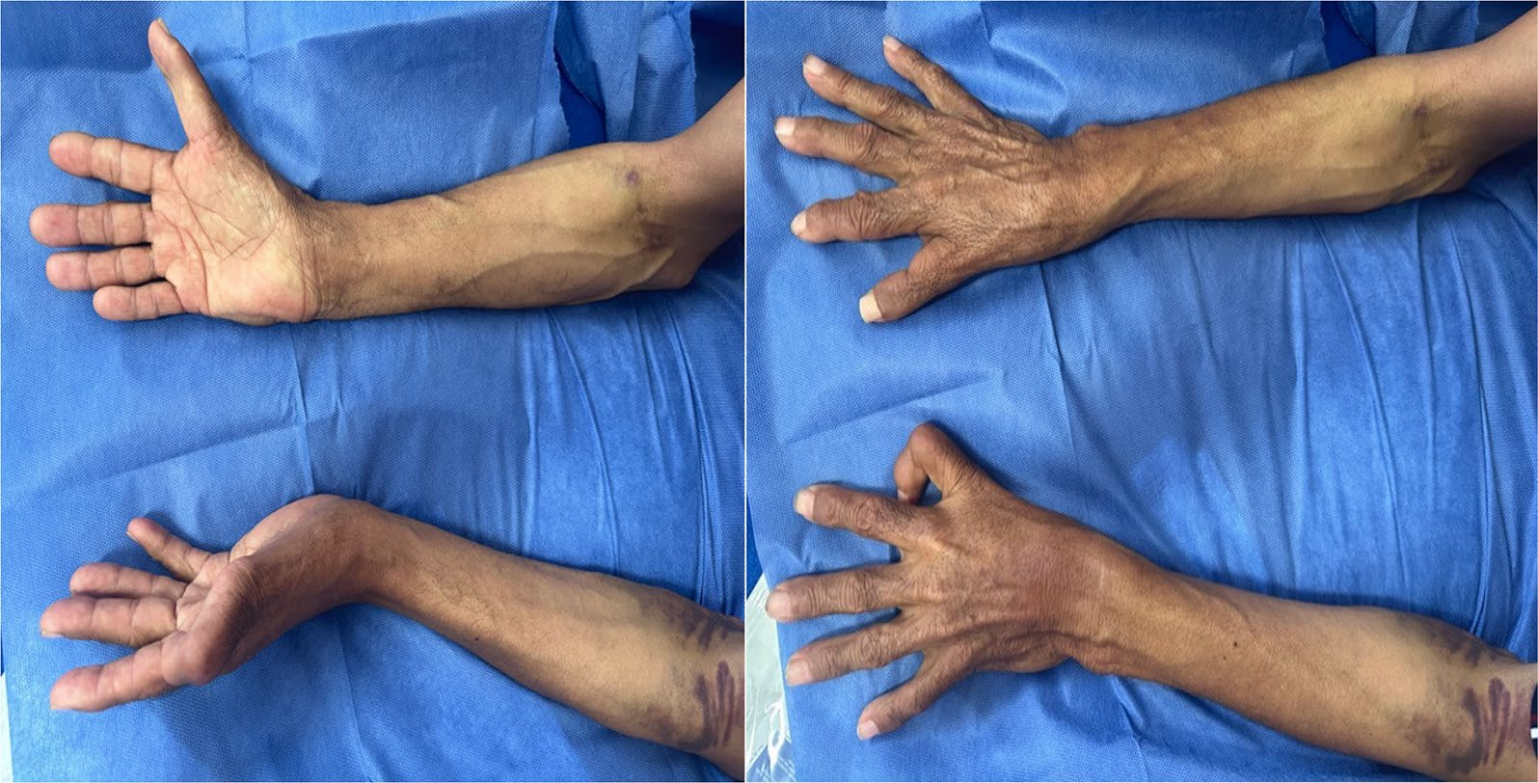
Photo showing the lack of prono-supination in the two forearms

Clinical examination also revealed a triphalangeal (Fig. 1) left thumb (red arrow), with the thumbs unable to oppose to other fingers, and a prono-supination defect affecting both forearms (Fig. 2).

**Fig. 3:**
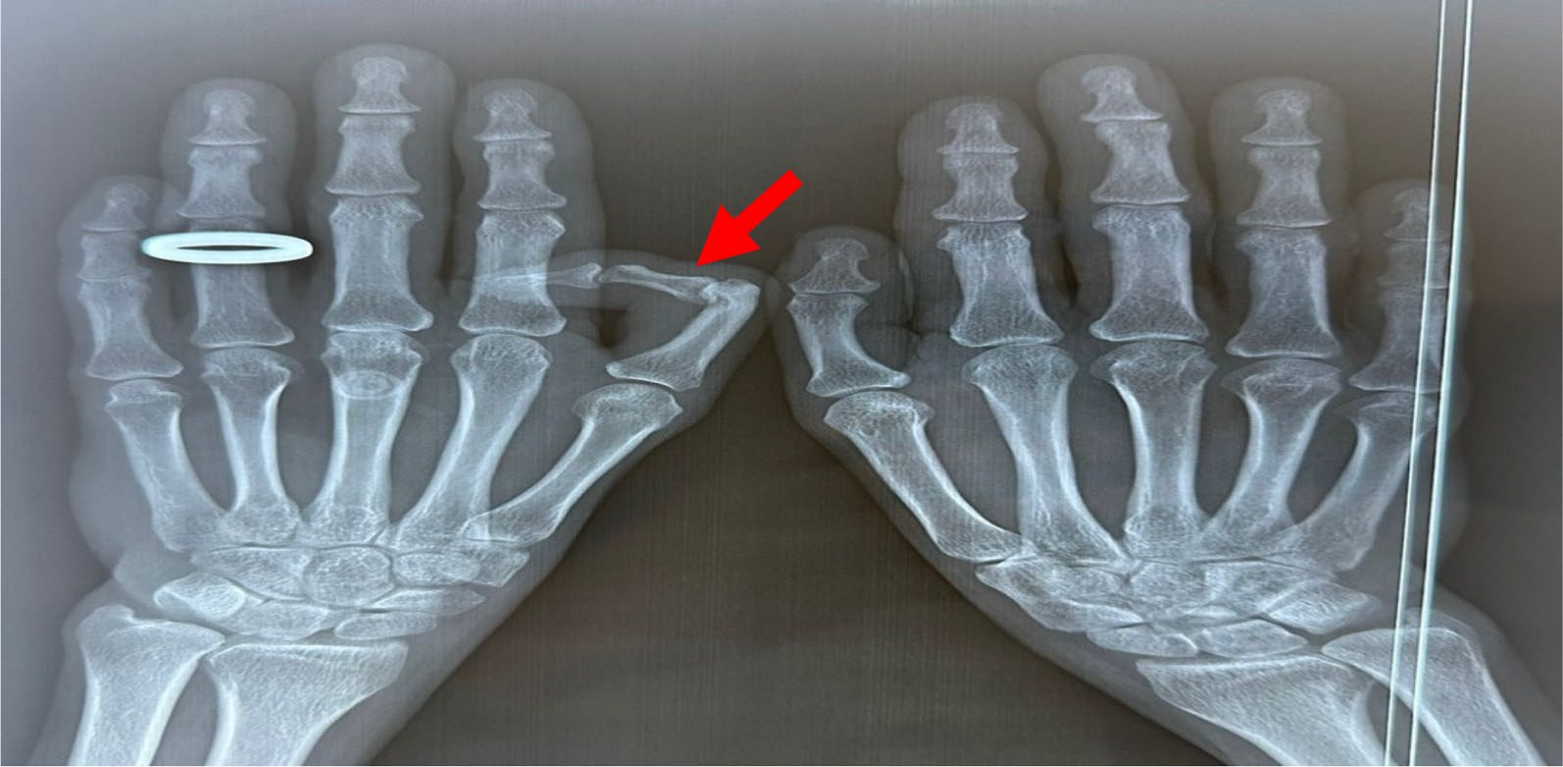
Hand X-rays confirming a triphalangeal left thumb (rogue arrow)

In view of this clinical anomaly, X-rays of both hands were taken (Fig. 3), confirming the triphalangeal left thumb (red arrow).

**Fig. 4:**
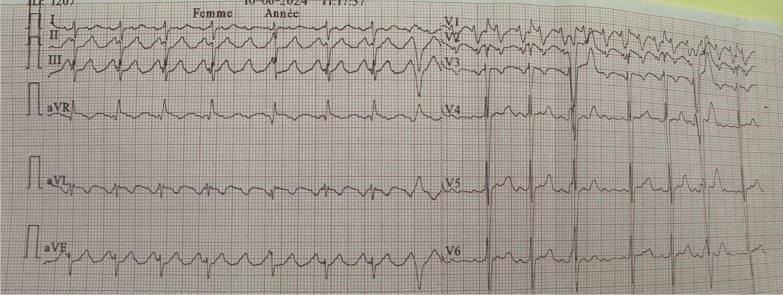
Patient's ECG showing atypical flutter

The ECG (Fig. 4) of our patient on admission showed an atypical 3/1 conduction atrial flutter at 84 bpm with ventricular extrasystoles.

**Fig. 5:**
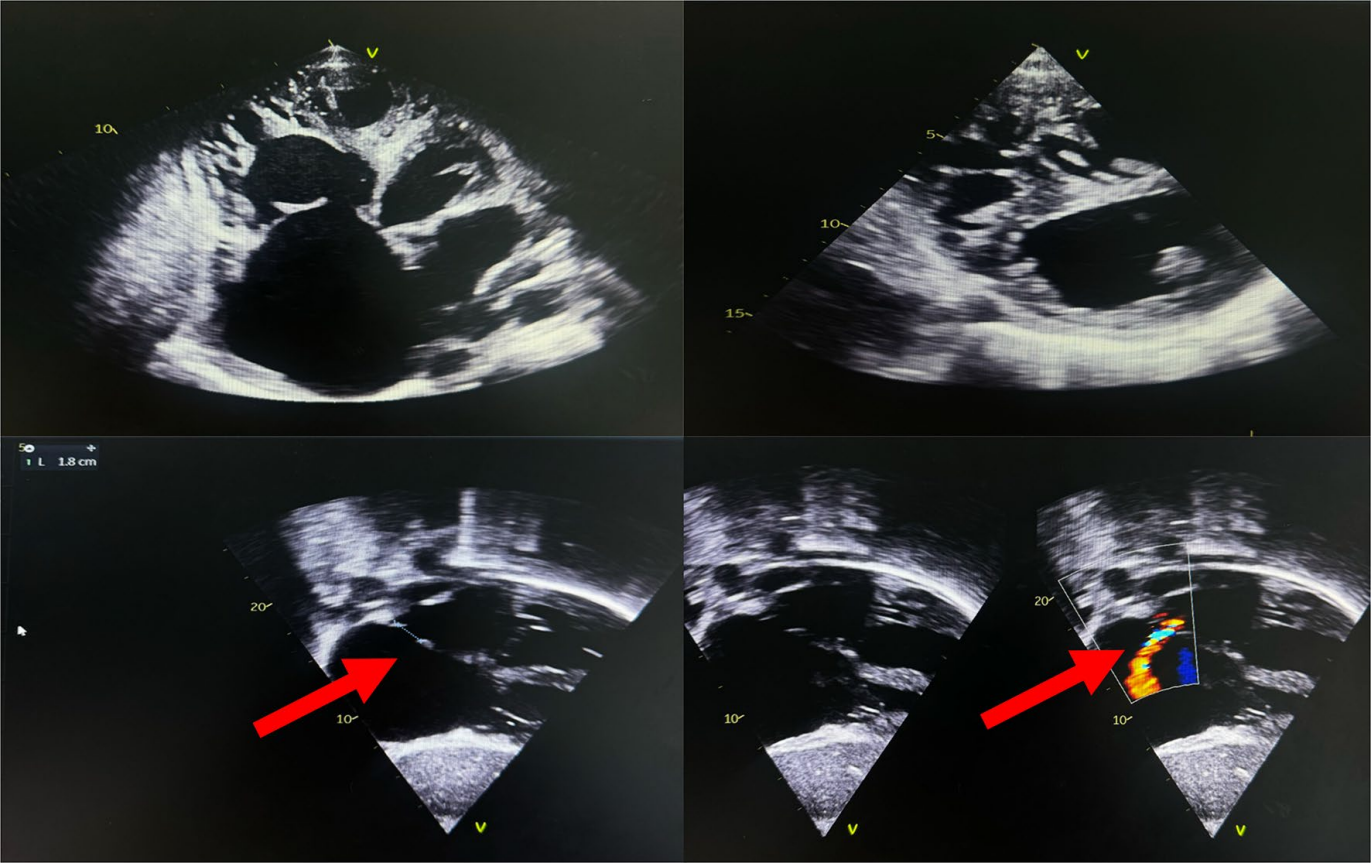
Transthoracic echocardiography showing interatrial communication ostium secondum (red arrows)

Transthoracic echocardiography (Fig. 5) revealed a left ventricle dilated to 33 mm/m^2^, with global hypokinesis, some trabeculations and an LVEF calculated at 40% by Simpson biplane. There was also an 18-mm ostium secondum interatrial communication and a left–right shunt (red arrows). The right cavities were extremely dilated, with a spider-webbed right ventricle, in longitudinal systolic dysfunction (TAPSE = 10 mm). The inferior vena cava was dilated to 26 mm and non-compliant, with pulmonary pressures estimated at 52 mmHg, in addition to minimal pericardial effusion. There was no significant mitroaortic valve disease, and the aorta was of normal caliber in the explored segments.

**Fig. 6:**
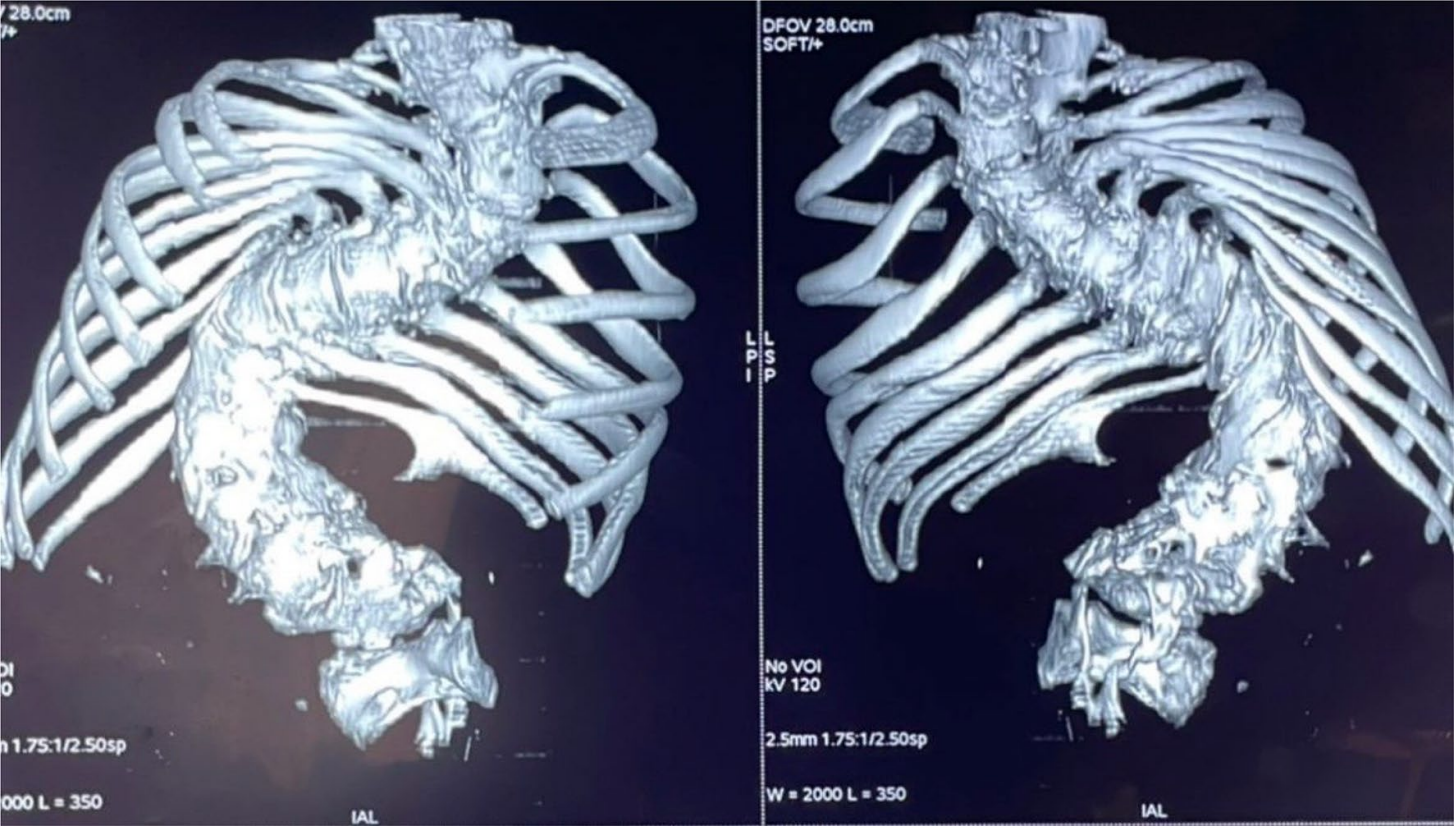
Spinal scan reconstruction confirming dorsolumbar scoliosis

In view of these anatomical anomalies and the presence of a spinal deformity, we ordered a thoracic X-ray, which showed scoliosis, and then supplemented this with a spinal CT scan with reconstruction (Fig. 6), confirming dorsolumbar scoliosis.

An abdominal ultrasound was performed to look for associated visceral malformations but came back with no abnormalities.

A genetic consultation was requested, and a genetic study was carried out, which revealed a mutation in the TBX5 gene, confirming Holt–Oram syndrome. Given that this syndrome is inherited in an autosomal dominant fashion, we carried out a family survey in search of similar cases, which proved negative.

Therapeutically, our patient was started on Furosemide injection for decongestion, in addition to potassium supplementation to prevent hypokalemia, with the addition of Ramipril 5 mg/day, Spironolactone 50 mg/day, followed by Bisoprolol 5 mg/day as a beta-blocker after decongestion and Dapagliflozin 10 mg/day. The evolution was marked by the patient's decongestion under intravenous diuretic treatment and then switch to oral treatment, slowing of the flutter under beta-blocker, and clear regression of dyspnea and palpitations under medical treatment.

For the LV dysfunction, a coronary angiography was carried out, revealing a normal, healthy coronary artery free of any significant lesion. In order to identify the cause of this LV dysfunction, a cardiac MRI was requested, but the patient did not have the means to have it done, given the unavailability of this technique in our public hospital.

For his scoliosis, the patient was referred to orthopedic surgery for specialized management. The patient was assessed by orthopedic surgeons, the decision was to schedule surgery for his scoliosis after stabilization of his heart condition and optimization of his heart failure treatment, a surgery that he refused.

As for his ASD, the patient refused all invasive procedures, whether right heart catheterization or percutaneous closure, and choose to stay under medical treatment alone.

The patient was then followed up in consultation for 6 months, once a week for the 1st month, twice a month for 2 months and then once every month for 3 months and until now, without any decompensation episode or rehospitalization, and with a clear improvement in quality of life on medical treatment alone, since he refused every other invasive therapy, neither for the ASD nor for the scoliosis.

## Discussion

Holt–Oram syndrome was first described in 1960 by Holt and Oram at King College Hospital in London [4]. The syndrome is very rare, autosomal dominant, with an incidence of around 0.95 per 100,000 births, with no predilection for either male or female sex [5].

The characteristics of this syndrome are anomalies of one or both upper limbs, with, more rarely, anomalies of the shoulder girdle, and often congenital cardiac malformations. The typical combination is considered to be a triphalangeal thumb with an intra-auricular communication secondum, but the severity of the cardiac and skeletal lesions can be highly variable and condition the prognosis [6].

The most frequent cardiac malformations are septal interatrial and/or interventricular septal defects, coarctation of the aorta with or without aortic bicuspidism, mitral valve anomalies, patent ductus arteriosus, conduction, and/or rhythm disorders [7]. Skeletal malformations of the upper limbs include thumb anomalies (absence or hypoplasia, triphalangia or syndactyly), agenesis or hypoplasia of the radius, ulna, or even humerus. Hypoplasia of the clavicles and anomalies of the thorax and/or spine may also be observed [8]. Our patient presents with a triphalangeal left thumb, fairly important dorsolumbar scoliosis, interatrial communication ostium secondum and an atrial flutter, in line with the literature and the historical description of this syndrome.

Vanlerberghe et al. [9] reported in their work that there are several genetic variants of this syndrome, also due to mutation of the TBX5 gene but with some variations on the molecular level, and manifested by other anomalies such as "pectus excavatum", pulmonary agenesis, Tetralogy of Fallot, or cardiomyopathy without septal defect.

According to the literature, these variants can be explained by the involvement of TBX5 in the development of the sternum and lung too and may be due to incomplete penetrance of this mutation [10–12].

The main differential diagnoses of SOH described by Vanlerberghe et al. [9] are Okihiro syndrome, Fanconi anemia, and TAR syndrome. For our patient, Holt and Oram syndrome was confirmed due to the presence of the TBX5 gene mutation, which allowed other differential diagnoses of this syndrome to be ruled out.

Silengo et al. [13] in turn described the Heart-Main syndrome type II or Tabatznik syndrome associating brachydactyly type D with supraventricular tachycardia, Ruiz de La Fuente and Prieto [14] described the Heart-Main Syndrome type III associating brachydactyly type C with sick sinus syndrome, while Hollister described the Long Thumb-Brachydactyly Syndrome [15]. Thus, there are many differential diagnoses of SOH and some may present similar clinical abnormalities, and the difference between all these different syndromes can only be made by genetic study.

In the absence of specific treatment for this condition, we resorted to the treatment of heart failure with reduced LVEF, including diuretics in case of congestive signs, a beta-blocker (Bisoprolol) for his arrhythmia and for the heart failure, an anti-mineralocorticoid (Spironolactone) and an SGLT2 inhibitor, based on the recommendations of the European Society of Cardiology for the management of heart failure [16]. This is just one case report, limited essentially by the number of patients, which is reduced to 1, and by the very limited financial resources available in a middle-income country.

## Conclusion

Holt–Oram syndrome is a rare genetic disorder that can have a number of clinical implications, including skeletal malformations affecting mainly the upper limbs in association with congenital heart defects, which in certain situations can be life-threatening. The syndrome should be suspected in the presence of any upper limb anomaly associated with a congenital heart defect and confirmed by genetic study. Once the diagnosis has been confirmed, a family investigation is required, given the autosomal dominant mode of transmission. To date, there is no specific treatment for this condition, and management will essentially be based on the treatment of skeletal malformations if indicated, and on the management of any cardiac pathologies expressed. With reference to our case report, we understand that every patient must be seen, assessed and examined as a whole, and that certain cardiac malformations, although frequent, may in certain situations fall into the category of rare syndromic diseases that require the most comprehensive patient management possible.

## Data Availability

All data and material to this report are accessible at any time upon request.

## Abbreviations

TBX5: T-box transcription factor 5
BPM: Beat per minute
ECG: Electrocardiogram
ASD: Atrial septal defect
LVEF: Left ventricle ejection fraction
TAPSE: Tricuspid annulus plane systolic excursion
CT: Computed tomography
TAR: Thrombocytopenia absent radius
SGLT2: Sodium–glucose cotransporter 2 inhibitor
LV: Left ventricle
